# Demonstration of 10 nm Ferroelectric Al_0.7_Sc_0.3_N-Based Capacitors for Enabling Selector-Free Memory Array

**DOI:** 10.3390/ma17030627

**Published:** 2024-01-27

**Authors:** Li Chen, Chen Liu, Hock Koon Lee, Binni Varghese, Ronald Wing Fai Ip, Minghua Li, Zhan Jiang Quek, Yan Hong, Weijie Wang, Wendong Song, Huamao Lin, Yao Zhu

**Affiliations:** Institute of Microelectronics, Agency for Science, Technology and Research (A*STAR), Singapore 138634, Singaporebinni_varghese@ime.a-star.edu.sg (B.V.); quek_zhan_jiang@ime.a-star.edu.sg (Z.J.Q.); song_wen_dong@ime.a-star.edu.sg (W.S.);

**Keywords:** AlScN, ferroelectric, capacitor, FeRAM, selector-free memory array

## Abstract

In this work, 10 nm scandium-doped aluminum nitride (AlScN) capacitors are demonstrated for the construction of the selector-free memory array application. The 10 nm Al_0.7_Sc_0.3_N film deposited on an 8-inch silicon wafer with sputtering technology exhibits a large remnant polarization exceeding 100 µC/cm^2^ and a tight distribution of the coercive field, which is characterized by the positive-up-negative-down (PUND) method. As a result, the devices with lateral dimension of only 1.5 μm show a large memory window of over 250% and a low power consumption of ~40 pJ while maintaining a low disturbance rate of <2%. Additionally, the devices demonstrate stable multistate memory characteristics with a dedicated operation scheme. The back-end-of-line (BEOL)-compatible fabrication process, along with all these device performances, shows the potential of AlScN-based capacitors for the implementation of the high-density selector-free memory array.

## 1. Introduction

Ferroelectric random-access memory (FeRAM), a promising non-volatile memory technology, has garnered significant research interest due to its fast read/write speed and low power consumption compared with other emerging memory technologies, such as resistive random-access memory (RRAM), magnetoresistive random-access memory (MRAM), and phase-change memory (PCM) [1]. However, most existing FeRAM arrays require selection devices, such as a transistor, to mitigate the read disturbance and prevent unintended programming [2,3,4]. The presence of selectors makes it more challenging for aggressive cell and array miniaturization, as well as multi-layer memory 3D stacking at complementary metal-oxide semiconductor (CMOS) back-end-of-line (BEOL). To address these issues, a novel architecture known as the selector-free FeRAM array was proposed [5]. In this architecture, given that the coercive voltage of the capacitors is *V*_c_, the memory window is defined as the ratio between the switching current (“on” state) and the non-switching current (“off” state) of the selected cell with a voltage of *V*_p_ applied, where *V*_p_/2 < *V*_c_ < *V*_p_. The current is then sensed by an operation amplifier and read as the voltage. The output voltage is proportional to the current and will be compared with a threshold value. However, we realized that the selector-free FeRAM array requires the ferroelectric material to possess a square-shaped *P*-*V* hysteresis loop with a large remnant polarization (*P*_r_) and a tight coercive field (*E*_c_) distribution to enable a low disturbance rate. If the hysteresis loop lacks sufficient squareness, the half-selected cell can generate unwanted disturbance signals due to the partial polarization switching.

The discovery of ferroelectricity in scandium-doped aluminum nitride (AlScN), which has a large remnant polarization >100 µC/cm^2^ and a steep switching slope (tight coercive voltage distribution) [6,7,8,9,10,11,12,13,14,15], shows its great potential to be implemented in selector-free FeRAM arrays. In addition, different synthesis methods have been developed to grow the wafer-scale AlScN, including the physical vapor deposition (PVD) [16,17,18,19,20,21], pulsed laser deposition (PLD) [22,23], and molecular beam epitaxy (MBE) [24,25]. The growth temperature of AlScN is below 400 °C, which satisfies the thermal budget of the CMOS BEOL integration. Furthermore, compared with element-doped HfO_2_ [26,27,28], which is another widely studied ferroelectric material family, there is no requirement for high temperature post annealing to obtain the ferroelectric AlScN. To date, some ferroelectric AlScN-based memory devices have been reported. For example, the researchers from the University of Pennsylvania successfully demonstrated a AlScN/two-dimensional channel materials-based ferroelectric field-effect transistor (FeFET) for in-memory computing [20,21]. The large memory window was achieved in the reported devices resulting from the large remnant polarization in AlScN film. However, the abovementioned desired features have to remain in the AlScN film down to 10 nm or thinner in order to be practical for memory applications. More recently, reports have shown that the AlScN can obtain ferroelectricity with a thickness of less than 10 nm [29,30,31]. Nevertheless, the reported ultra-thin AlScN is either required to be grown on the GaN substrate, which is not compatible with the CMOS BEOL integration [31], or the devices must be based on simple test structures with a big size (i.e., metal–ferroelectric–metal pillars with a global bottom electrode), which might cause large power consumption owing to a large leakage current [29,30,31]. Additionally, the integration of passivated devices enabling the crossbar array architecture has not been demonstrated yet.

In this case, ultra-thin AlScN-based passivated FeRAM devices on silicon substrates with a small size need to be examined. In this work, we experimentally demonstrate a 10 nm thick Al_0.7_Sc_0.3_N-based passivated capacitor with CMOS BEOL compatibility for constructing a selector-free array for memory applications. The ferroelectricity is observed in 10 nm AlScN with a 2*P*_r_ larger than 200 µC/cm^2^ and a switching speed faster than 50 ns. The device shows a memory window larger than 250%, a low power consumption of ~40 pJ, and a low disturbance rate (*R*_d_) smaller than 2%. In addition, the devices exhibit the multistate memory property, indicating the potential application for in-memory computing.

## 2. Experiment and Method

**Figure 1:**
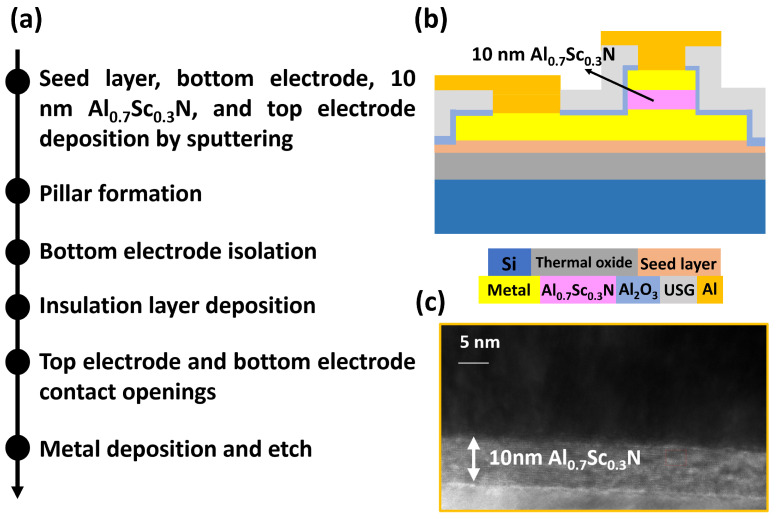
(**a**) Fabrication process flow and (**b**) structure of the 10 nm Al_0.7_Sc_0.3_N-based FeRAM device. (**c**) TEM image of the top electrode and AlScN stacks, confirming that the thickness of Al_0.7_Sc_0.3_N is 10 nm.

Figure 1a shows the process flow for realizing a 10 nm Al_0.7_Sc_0.3_N-based passivated capacitor on an 8-inch silicon (Si) substrate. Firstly, the seed layer, bottom electrode, 10 nm Al_0.7_Sc_0.3_N layer, and top electrode are deposited using the alloy target-based sputtering on the 8-inch thermal oxide/Si substrate without a vacuum break. Then, circular pillars with different feature sizes are formed by patterning and dry etching. The bottom electrodes are subsequently isolated using dry etching, followed by insulation layer deposition. Finally, the via holes are opened, and an aluminum (Al) layer is deposited and etched for routing and probe contacting. Figure 1b plots the structure of the passivated capacitor. The thermal budget of all process steps is below 400 °C, showing compatibility with future CMOS BEOL integration. Figure 1c shows the cross-sectional transmission electron microscope (TEM) image of the top electrode and AlScN stacks region of the device, where the thickness of AlScN is confirmed to be 10 nm.

## 3. Results and Discussion

**Figure 2:**
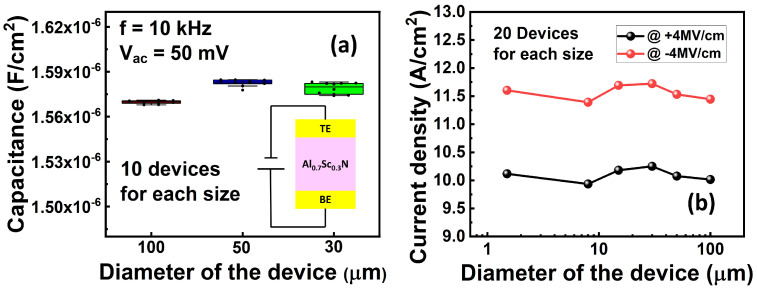
(**a**) Normalized capacitances of the devices with different sizes, showing size-independent behavior. The relative permittivity is extracted to be ~18. (**b**) DC currents of the devices with different sizes at ±4 MV/cm, showing size-independent behavior.

**Figure 3:**
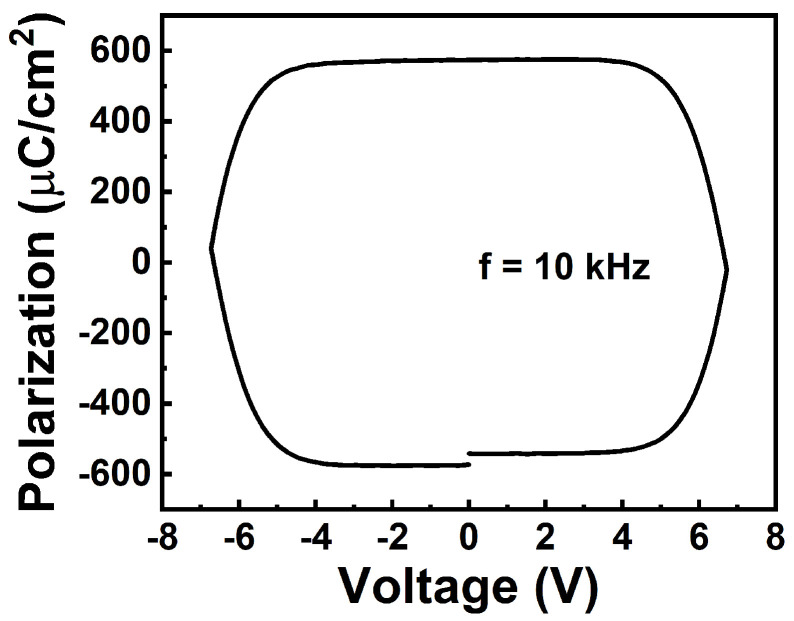
Non-PUND *P*-*V* curve of the 10 nm Al_0.7_Sc_0.3_N-based capacitor. The *P*_r_ value is due mainly to the leakage current.

**Figure 4:**
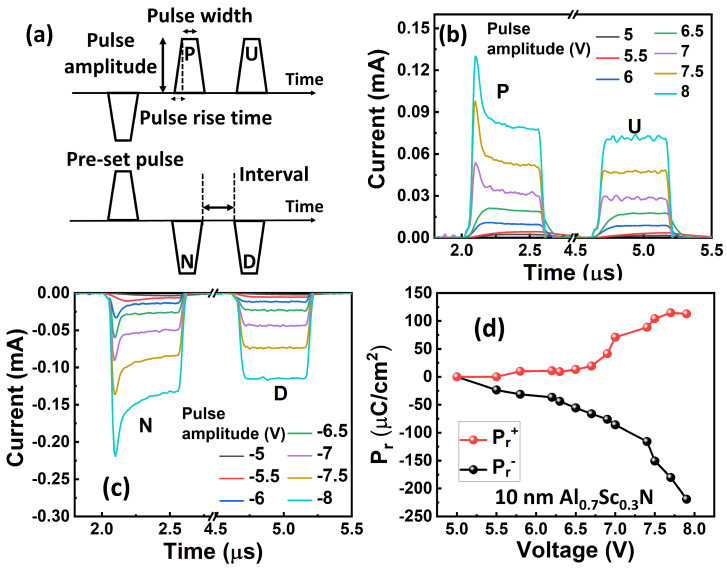
(**a**) Schematic of the PUND method for characterizing the ferroelectricity of 10 nm Al_0.7_Sc_0.3_N film. Transient current response to the (**b**) “P” & “U” pulses and (**c**) “N” & “D” pulses. Ferroelectricity-induced switching current is clearly observed in “P” and “N” pulses. (**d**) Extracted *P*_r_ vs. pulse amplitude shows that 10 nm Al_0.7_Sc_0.3_N obtains *P*_r_ larger than 100 µC/cm^2^.

Figure 2 shows the measurement results of the normalized capacitance (Figure 2a) and DC leakage current density (Figure 2b) at ±4 MV/cm of the isolated devices with different lateral sizes. The relative dielectric permittivity of the 10 nm Al_0.7_Sc_0.3_N is extracted to be ~18, which is in agreement with the literature [32]. The leakage current density is ~10 A/cm^2^ at ±4 MV/cm. Additionally, both the capacitance and leakage current density show size-independent behavior, which indicates that the size can be further scaled down to reduce the leakage current. In this work, all electrical testing was performed with the voltage applied to the bottom electrode, while the top electrode was grounded. Owing to the relatively large leakage current, the *P*_r_ value obtained from the traditional *P*-*V* measurement as shown in Figure 3 is due primarily to the leakage current, and the intrinsic ferroelectric behavior cannot be identified fairly. Instead, we further used the PUND method for characterizing the ferroelectricity of 10 nm Al_0.7_Sc_0.3_N, as shown in Figure 4a. A pre-set pulse with an amplitude of −8 V/+8 V is applied, followed by two consecutive pulses with opposite pulse amplitudes, named “P” & “U” (or “N” & “D”, depending on the pulse polarity). The pulse rise time, pulse width, and interval of applied pulse are 50 ns, 500 ns, and 2 µs, respectively, in this work if not specifically mentioned. Figure 4b,c shows the transient current responses to the “P” & U” pulses and “N” & “D” pulses of a 1.5 µm diameter capacitor with various amplitudes ranging from 5 V to 8 V. It shows clear ferroelectricity-induced polarization switching currents when the pulse amplitude of “P” and “N” are larger/smaller than 7 V/−6 V, respectively. The *P*_r_ is extracted to be >100 µC/cm^2^ via the subtraction of the non-switching current in “U” and “D”, exhibiting no obvious degradation compared with thicker films [19], as shown in Figure 4d. It should be noted that the *P*_r_ at the negative side does not saturate because the leakage current cannot be fully compensated when the applied voltage is large, even though the PUND method is used. The extra *P*_r_ is due to the uncompensated leakage current. Therefore, the *P*_r_ value that was extracted using the PUND method is still a little overestimated, especially at the high voltage. The memory window is defined as the ratio between the peak of switching current in the “P” or “N” pulse and the non-switching current in the “U” or “D” pulse. Additionally, there is a narrow distribution of *E*_c_ in which the polarization starts to switch at 7 V or −6 V and completes switching at 7.5 V or −7 V for positive or negative switching, respectively, and there is almost no polarization switching when a voltage smaller than the coercive voltage is applied, indicating its potential to be implemented in the selector-free memory array.

**Figure 5:**
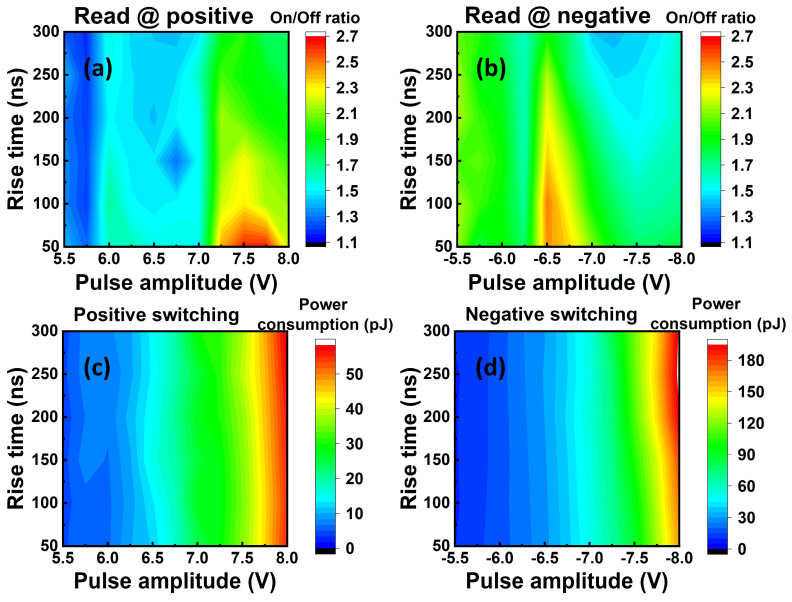
Heatmap of the on/off ratio and power consumptions with different pulse rise time and pulse amplitudes. (**a**,**b**) On/off ratio of the device using positive and negative pulses to read. Power consumption of the device under (**c**) positive switching and (**d**) negative switching operation modes. The power consumption of the device under optimal conditions is ~40 pJ. The size of measured device is 1.5 µm in diameter.

**Figure 6:**
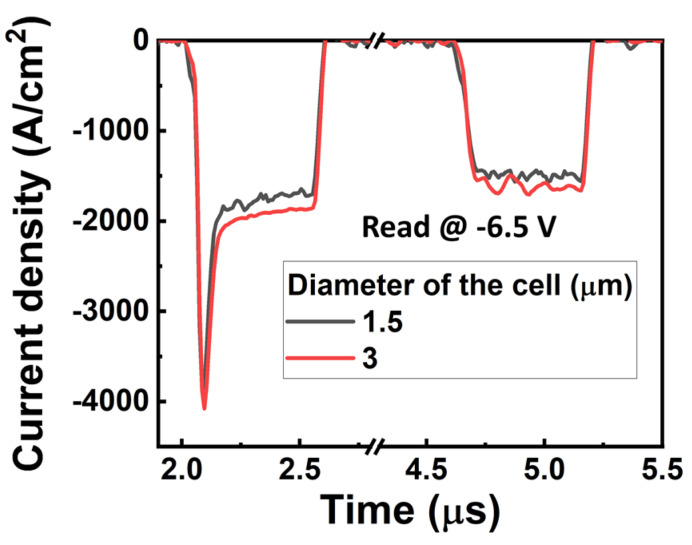
Transient response of the devices with different sizes. The size of device shows negligible impact on the on/off ratio.

**Figure 7:**
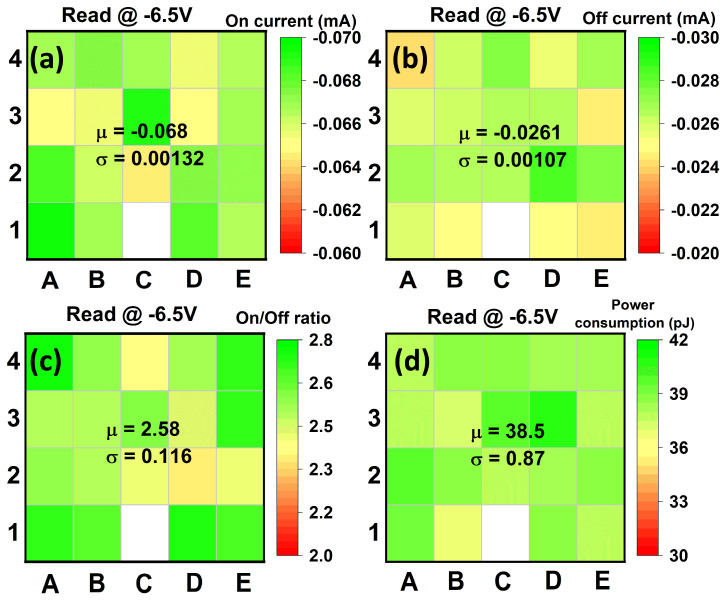
Heatmap of the (**a**) on current, (**b**) off current, (**c**) on/off ratio, and (**d**) power consumption of the 5 × 4 selector-free array read at −6.5 V. The devices show good uniformity across the array. The white area refers to the short device, and the cell size is 1.5 µm in diameter. The letters “1”—“4” and “A”—“E” refer to the vertical and horizontal location of the devices in the array, respectively.

Then we examined the optimal operation modes of our device by tuning the pulse rise time and pulse amplitude for both positive and negative reading modes. Under the positive reading mode, we wrote the “on” state by applying a negative pre-set pulse, while we wrote the “off” state by applying a pre-set positive pulse for the writing operation. For the reading operation under the positive reading mode, the unknown state could be read by applying a positive pulse. If the initial state is “off”, we would obtain a low non-switching current. On the contrary, if the initial state is “on”, we would obtain a high switching current, and subsequently, another negative pre-set pulse is required to reset the state to the “on” state. On the other hand, under the negative reading mode, we wrote the “on” state by applying a positive pre-set pulse, while we wrote the “off” state by applying a pre-set negative pulse for the writing operation. For the reading operation under the negative reading mode, the initial state could be identified by applying a negative pulse. If the initial state is “off”, there would be a non-switching current; if the initial state is “on”, there would be a high switching current, and a positive pre-set pulse is needed for refreshing the state to the “on” state. Figure 5 shows the results of the device in terms of memory window and power consumption under different reading conditions. The highest memory window of ~250% is obtained at ~7.5 V and ~−6.5 V, with a pulse rise time of 50 ns for the positive and negative reading modes (Figure 5a,b), respectively. These results show a clear trend that a shorter pulse rise time enables a larger memory window. It should be noted that the memory window can be further increased by reducing the leakage current of the AlScN using strain engineering [11,12] in which the non-switching current can be effectively reduced with a neutral strain. Due to the fast switching speed, the power consumption under these operation modes is ~40 pJ (Figure 5c,d), which is comparable to the representative crossbar arrays, although the operation voltage is high [33,34,35]. Additionally, owing to the behavior of the size-independent leakage current, the transient response also shows the size-independent characteristic, meaning that the device size will not affect the on/off ratio, as shown in Figure 6. This indicates that the power consumption can also be reduced by reducing the device size. Subsequently, we examined the performance of a 5 × 4 selector-free array under the optimal operation mode, as shown in Figure 7, in which the negative reading mode was employed. The selector-free array shows a high yield of 95% and good uniformity. The average current levels of the “on” state (Figure 7a), “off” state (Figure 7b), memory window (Figure 7c), and power consumption (Figure 7d) are ~0.068 mA, ~0.026 mA, ~258%, and 38.5 pJ, respectively. Their corresponding coefficients of variation (σ/µ) are 1.9%, 4%, 4.5%, and 2.3%, respectively. The good uniformity indicates that the array density could be scaled up further.

**Figure 8:**
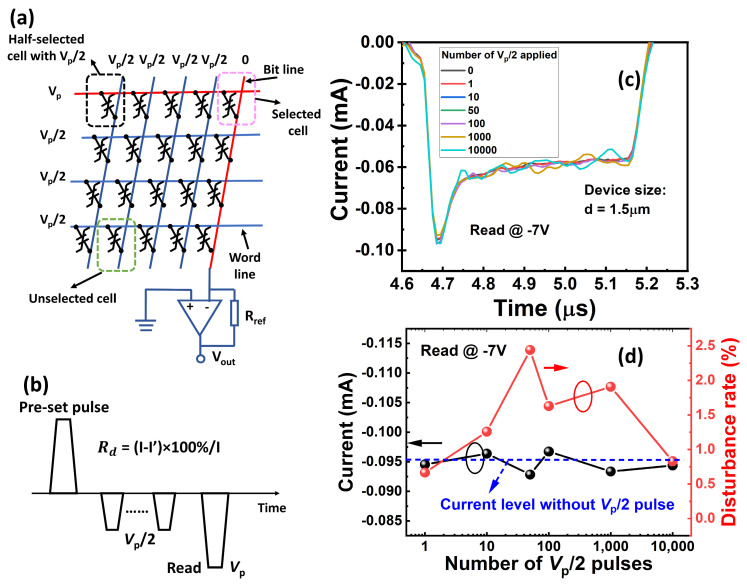
(**a**)Schematic of the selector-free array architecture based on ferroelectric FeRAM devices. (**b**) Schematic of measuring the current response after the *V*_p_/2 (**c**) The transient current response of switching current after applying different numbers of *V*_p_/2 pulses, showing no obvious degradation. (**d**) Extracted switching current and disturbance rate. The current remains the same level after applying numbers of *V*_p_/2 pulses, and the disturbance rate is <2%.

**Figure 9:**
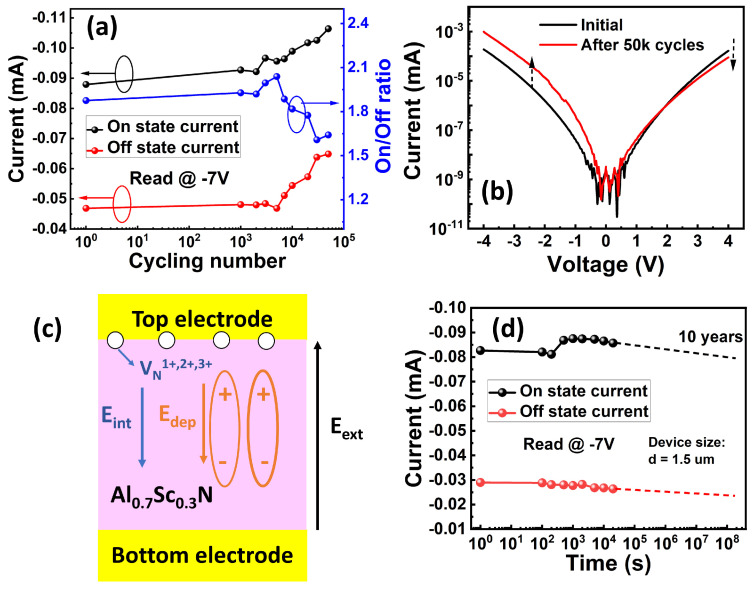
(**a**) Endurance behavior of the FeRAM device. (**b**) The DC *I-V* curves of the device after cycling, showing that the negative leakage current increases while the positive leakage current decreases. (**c**) Schematic illustration of the reduced positive leakage current. (**d**) Retention behavior of on and off current states reading at −7 V shows up to 20,000 s in measurement and 10-year extrapolation with a high memory window.

In a selector-free array, the cells that are located at the same word line or bit line with the target cell are half-selected and applied with *V*_p_/2 during the reading or writing operations, as illustrated in Figure 8a. It is essential to check whether these *V*_p_/2 pulses would impact the polarization state when the device is half-selected. Figure 8b shows a schematic that outlines the method used to examine the disturbance under negative reading mode. Firstly, we used a −7 V (*V*_p_) pulse to read the switching current (*I*) of the initial state with a pre-set pulse of 8V. Then, before reading the current (*I’*), we reset the state and applied a series of −3.5 V (*V*_p_/2) pulses using a negative pulse with a pulse amplitude of −7 V to mimic the half-selected situations. There is negligible variation between *I* and *I’*, and the disturbance rate (*R*_d_ = *(I* − *I’)/I*) is extracted to be <2% with 10,000 *V*_p_/2 pulses, as shown in Figure 8c,d. This low disturbance rate results from the steep switching slope (i.e., narrow distribution of *E*_c_) of Al_0.7_Sc_0.3_N in which there is almost no polarization switching when a *V*_p_/2 pulse is applied. The excellent immunity to disturbance indicates its great potential for implementation in selector-free FeRAM arrays for memory applications. This finding agrees with the conclusion of our previous work in which both unipolar cycling with an applied voltage larger than the coercive voltage and bipolar cycling with an applied voltage smaller than the coercive voltage would not affect the devices [23]. In addition, the endurance characteristic was evaluated, as plotted in Figure 9a. The cycling pulses are square pulses with a pulse width of 500 ns and a pulse amplitude of 7 V/−6.8 V, which enables the polarization to be fully switched during the cycling. The tested device demonstrates an endurance exceeding 5 × 10^4^ before breakdown. However, under the negative reading mode, the reading current increases while the memory window remains above 160% after the cycling. The DC leakage current measurement shows a similar trend in which the positive leakage current decreases, while the negative leakage current increases, indicating the formation of an internal field that points from the top electrode to the bottom electrode after the cycling, as shown in Figure 9b. We suspected that nitrogen vacancies (*V*_N_) with positive charge states accumulate at the interface between the top electrode and the Al_0.7_Sc_0.3_N layer during the cycling because of the asymmetric cycling pulses, leading to the formation of an internal field (Figure 9c). This results in a decreased positive leakage current and pins the domains to flip.

Additionally, the retention performance of the device was tested. It maintains a large memory window throughout the entire 20,000 s measurement period, and it is reasonable to extrapolate the retention to reach the 10-year mark without notable degradation, as shown in Figure 9d.

**Figure 10:**
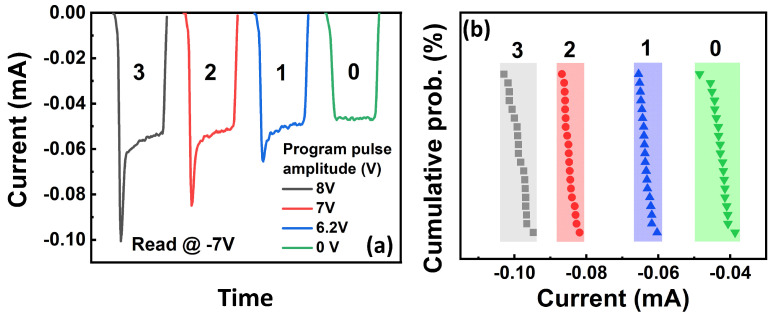
(**a**) Multistate switching behavior of the devices via programming with different pulse amplitudes. Multiple states, such as “0”, “1”, “2”, and “3”, are achieved by applying pre-set pulse with pulse amplitudes of 0, 6.2, 7, and 8 V, respectively. (**b**) Statistic result of multistate behavior of the devices, showing the clear gap between each memory state.

Finally, the FeRAM device exhibits multiple switching currents via programming with dedicatedly designed pulse amplitudes to induce partial polarization switching, as plotted in Figure 10a. Four distinct states are obtained by programming the devices with a pre-set pulse with pulse amplitudes of 0, 6.2, 7, and 8 V and reading at −7 V. There were 20 devices measured, and they showed no overlap between each state, as shown in Figure 10b, indicating its great promise to be used for in-memory computing.

Compared with other reported element-doped and hafnium-doped FeRAM arrays [4,36], which are based on a one-transistor–one-capacitor (1T1C) structure, the AlScN-based FeRAM has the advantage of being high-density because of the non-selector architecture and a comparable switching speed of <50 ns. However, the study of ferroelectric AlScN is still in the early stages, and it suffers from a high coercive field, large leakage, and relatively poor endurance. To enhance the performance of the AlScN-based FeRAM device, the leakage current and coercive field need to be further improved, which might be optimized using strain engineering [11].

## 4. Conclusions

In summary, we successfully demonstrate a 10 nm Al_0.7_Sc_0.3_N-based isolated FeRAM device for building up the selector-free FeRAM array. The whole process takes place below 400 °C, which is compatible with CMOS BEOL integration. The large *P*_r_ and steep switching slope (narrow *E*_c_ distribution) are obtained in the ultra-thin Al_0.7_Sc_0.3_N film, enabling the device to work with a large memory window, a low power consumption, and a low *R*_d_. In addition, the devices show no size-dependent behavior, indicating the potential to further reduce the power consumption by scaling down the device size. Furthermore, the multistate memory behavior achieved in the devices shows the potential to be implemented in this high-density architecture for in-memory computing.

## Data Availability

Data are contained within the article.
